# Factors associated with malaria parasitaemia, malnutrition, and anaemia among HIV-exposed and unexposed Ugandan infants: a cross-sectional survey

**DOI:** 10.1186/1475-2875-11-432

**Published:** 2012-12-27

**Authors:** Beth Osterbauer, James Kapisi, Victor Bigira, Florence Mwangwa, Stephen Kinara, Moses R Kamya, Grant Dorsey

**Affiliations:** 1 Department of Medicine, University of California, San Francisco General Hospital, 1001 Potrero Avenue, Building 30, Room 3420, San Francisco, CA, 94110, USA; 2 Infectious Diseases Research Collaboration, Kampala, Uganda; 3 Department of Medicine, Makerere University College of Health Sciences, Kampala, Uganda

## Abstract

**Background:**

Malaria, malnutrition and anaemia are major causes of morbidity and mortality in African children. The interplay between these conditions is complex and limited data exist on factors associated with these conditions among infants born to HIV-uninfected and infected women.

**Methods:**

Two hundred HIV-exposed (HIV-uninfected infants born to HIV-infected mothers) and 400 HIV-unexposed infants were recruited from an area of high malaria transmission in rural Uganda. A cross-sectional survey was performed at enrolment to measure the prevalence of malaria parasitaemia, measures of malnutrition (z-scores <2 standard deviations below mean) and anaemia (haemoglobin <8 gm/dL). Multivariate logistic regression was used to measure associations between these conditions and risk factors of interest including household demographics, malaria prevention practices, breastfeeding practices, household structure and wealth index.

**Results:**

The prevalence of malaria parasitaemia was 20%. Factors protective against parasitaemia included female gender (OR = 0.66, p = 0.047), mother’s age (OR = 0.81 per five-year increase, p = 0.01), reported bed net use (OR = 0.63, p = 0.03) and living in a well-constructed house (OR = 0.25, p = 0.01). Although HIV-unexposed infants had a higher risk of parasitaemia compared to HIV-exposed infants (24% *vs* 14%, p = 0.004), there was no significant association between HIV-exposure status and parasitaemia after controlling for the use of malaria preventative measures including bed net use and trimethoprim-sulphamethoxazole prophylaxis. The prevalence of stunting, underweight, and wasting were 10%, 7%, and 3%, respectively. HIV-exposed infants had a higher odds of stunting (OR = 2.23, p = 0.005), underweight (OR = 1.73, p = 0.09) and wasting (OR = 3.29, p = 0.02). The prevalence of anaemia was 12%. Risk factors for anaemia included older infant age (OR = 2.05 per one month increase, p = 0.003) and having malaria parasitaemia (OR = 5.74, p < 0.001).

**Conclusions:**

Compared to HIV-unexposed infants, HIV-exposed infants had a higher use of malaria preventative measures and lower odds of malaria parasitaemia. Having a better constructed house was also protective against malaria parasitaemia. HIV-exposure was the primary risk factor for measures of malnutrition. The primary risk factor for anaemia was malaria parasitaemia. These findings suggest the need to better target existing interventions for malaria, malnutrition and anaemia as well as the need to explore further the mechanisms behind the observed associations.

## Background

Malaria, malnutrition, and anaemia are among the major causes of childhood morbidity and mortality in sub-Saharan Africa [1,2]. Not only are these disease entities of major public health importance, they may also be inter-related in terms of causality, severity of illness, and underlying associated factors. In 2009, malaria was estimated to account for 16% of all deaths in children under five years of age in Africa [3]. Malnutrition has been estimated to be a contributing factor in more than half of childhood deaths related to major infectious diseases, including malaria [3,4]. Malnutrition has also been shown to influence both the manifestation of and susceptibility to malaria [5-7]. Malaria and malnutrition are causally associated with anaemia and underlying anaemia may contribute to the severity of malaria and malnutrition [6].

African infants under one year may be at particularly high risk of malaria, malnutrition and anaemia, both in terms of the prevalence and severity of these illnesses [2]. Adding to the complexity of this high-risk group is a rising population of HIV-exposed but uninfected infants born to HIV-infected mothers, due in large part to the successful implementation of prevention of mother-to-child transmission (PMTCT) programmes in Africa [7]. Exposure to HIV may have a direct or indirect role on child health and limited data exist on the prevalence and factors associated with malaria, malnutrition, and anaemia in this novel population. For example, infants born to HIV-infected mothers may have better access to health care services compared to infants born to HIV-uninfected mothers. Alternatively, infants born to HIV-infected mothers may have excess morbidity due to maternal HIV-related illness, exposure to opportunistic infections, the mother’s ability to breastfeed, or socioeconomic factors [8]. A better understanding of factors associated with malaria, malnutrition and anaemia in African infants both exposed and unexposed to HIV could help elucidate their complex relationship and consequently inform the design of interventions aimed at reducing disease burden.

To address some of these issues a cross-sectional survey was performed at enrolment in a cohort of HIV-exposed and unexposed infants living in a rural area of Eastern Uganda with high malaria transmission intensity and high prevalence of malnutrition [9,10]. The goals of this study were to describe the prevalence of malaria, malnutrition, and anaemia, examine the relationship between these disease entities, and generate plausible hypotheses regarding variables that could possibly be associated with these outcomes, including HIV exposure status.

## Methods

### Description of study site

The study was conducted in Tororo District, a rural area in Eastern Uganda characterized by subsistence farming and relatively high rates of poverty [6]. Malaria transmission in this area is very high and occurs perennially with an estimated entomological inoculation rate of 562 infective bites per person-year [9]. According to the 2006 Ugandan Demographic and Health Survey, 36% of children under the age of five in this region were classified as stunted and 72% were classified as anaemic, defined as a haemoglobin level <10 gm/dL [6]. In 2002, the UNAIDS sentinel survey found 6.3% of pregnant women in Tororo district to be HIV-infected [11].

### Study design, selection of study participants and data collection

This study was comprised of a cross-sectional survey done at the time of enrolment for a cohort of HIV-exposed and unexposed infants involved in an intervention trial evaluating different anti-malarial chemoprevention regimens. Between June 2010 and July 2011, children four to six months of age were recruited using convenience sampling at a dedicated study clinic located at Tororo District Hospital. Potential study subjects were recruited from an adjacent antenatal clinic and surrounding government health centres. Study selection criteria included the following: 1) confirmed HIV status of the biological mother, 2) negative HIV DNA PCR test at the time of enrolment for infants born to HIV-infected mothers, 3) residency within a 30-km radius of the study clinic, 4) agreement to come to the study clinic for any febrile episode or other illness and avoid medications given outside the study protocol, 5) not living in the same household as a previously enrolled participant, 6) absence of any active medical problem requiring inpatient evaluation or condition requiring frequent medical attention at the time of screening, and 7) provision of informed consent by the parent/guardian.

On the day of enrolment a standardized history and physical examination was conducted including single measurements of weight and length. Weight was measured to the nearest 0.1 kilogram using a digital Seca 354 scale and length was measured to the nearest centimeter using a recumbent length board. A questionnaire was also administered to the study subject’s primary caregiver that collected information on household demographics, bed net use, chemoprophylaxis, current breastfeeding practices, characteristics of the household structure, and household possessions.

### Laboratory methods

Blood was collected by phlebotomy for malaria smears and haemoglobin measurement. Thick blood smears were done on all study participants and thin smears were done on only study participants with symptomatic malaria (defined as tympanic temperature ≥ 38.0°C or history of fever in the last 24 hours and a positive thick blood smear). Thick and thin blood smears were stained with 2% Giemsa and read by experienced laboratory technologists who were not involved in direct patient care. A blood smear was considered negative when the examination of 100 high power fields did not reveal asexual parasites. For quality control, all slides were read by a second microscopist and a third reviewer settled any discrepant readings. Out of 600 thick blood smears read, 8 (1.3%) had discrepant readings with regards to whether malaria parasitemia was present, requiring resolution by a third reader. Haemoglobin levels were measured using a Beckman Coulter machine.

### Statistical analysis

Data collected on the day of enrolment were recorded on standardized case record forms and double entered into a Microsoft Access database. Data analysis was conducted using STATA version 12.1 (STATA Corp., College Station, TX, USA).

Predictor variables of interest included household demographics, malaria prevention practices, breastfeeding practices, household structure and wealth index. A well-constructed house was defined as reported to have iron sheets on the roof, burnt bricks or cement walls, and a cement floor. Wealth was measured by the reported ownership of household items such as telephone, radio and bicycle among others, that were then aggregated in a principal component analysis and categorized into lowest, middle and highest tertile of household wealth index. Outcomes of interest included 1) malaria parasitaemia, defined as the presence of any asexual parasites, 2) measures of malnutrition (stunted, underweight, and wasted) defined as a z-scores <2 standard deviations below the mean length-for-age, weight-for-age, and weight-for-length z-scores, respectively, as per the WHO Global Database on Child Growth and Malnutrition and 3) moderate to severe anaemia, defined as a haemoglobin level <8 gm/dl.

Comparisons of characteristics between HIV-exposed and unexposed infants were made using the Chi-squared test for categorical variables and t-test for continuous variables. Associations between predictor variables and outcomes of interest were estimated using both univariate and multivariate logistic regression with HIV exposure status included as a covariate. Predictor variables with a p-value <0.10 using univariate analyses were reported and included in the initial multivariate models. Final multivariate models were generated using backwards selection with the final models retaining only those variables with a p-value <0.10 or a strong biological plausibility for association. In the final interpretation of results a p-value <0.05 was considered statistically significant without adjustment for multiple comparisons.

## Results

### Characteristics of study population and comparison of HIV exposure groups

Of the 600 participants enrolled, 200 were HIV-exposed (HIV-uninfected children born to HIV-infected mothers) and 400 were HIV-unexposed (HIV-uninfected children born to HIV-uninfected mothers) based on the target sample size for the parent clinical trial. Comparisons of HIV-exposed and HIV-unexposed children are presented in Additional file 1. The median age at enrolment was 5.2 months, and HIV-exposed children were slightly younger compared to HIV-unexposed children (4.8 *vs* 5.4 months, p < 0.001). HIV-exposed children had significantly older mothers (30.7 *vs* 25.5 years, p < 0.001) and a higher proportion of their biological fathers were deceased (8% *vs* 1%, p < 0.001).

A higher proportion of HIV-exposed children reported using malaria preventative measures including the use of any bed nets, insecticide-treated nets (ITNs), and trimethoprim-sulphamethoxazole (TS) prophylaxis (p < 0.001 for all comparisons), although reported use of TS prophylaxis was relatively low (15%) among HIV-exposed children despite this being a national recommendation. A higher proportion of mothers of HIV-exposed children reported the use of any bed nets and ITNs, both currently and during their last pregnancy (p < 0.001 for all comparisons). Consistent with recommendations for all HIV-infected patients in Uganda, the mothers of HIV-exposed children reported >90% coverage with TS prophylaxis both currently and during their last pregnancy. A higher proportion of mothers of HIV-unexposed children reported taking sulphadoxine-pyrimethamine (SP) prophylaxis during their last pregnancy, but the reported use of SP was still high among the mothers of HIV-exposed children (81% *vs* 55%) despite the fact that co-administration of TS and SP is not recommended during pregnancy. Over 99% of children were currently breastfeeding; however, a higher proportion of HIV-exposed children were exclusively breastfeeding while a higher proportion of HIV-unexposed children were partially breastfeeding. Measures of household wealth were similar between the two HIV exposure groups, but a higher proportion of HIV-exposed children lived in better constructed houses characterized by roofs with iron sheets, burnt brick or cement walls, and cement floors.

### Predictor variables associated with malaria parasitaemia

Considering all 600 HIV-exposed and unexposed study participants, 122 (20.3%) had a thick blood smear positive for malaria parasitaemia. Among those with parasitaemia, 47 (38.5%) had symptomatic malaria. Thin blood smears performed only on those with symptomatic malaria revealed 45 with *P. falciparum*, one with *Plasmodium malariae*, and one with *Plasmodium ovale*. Significant associations between predictor variables of interest and malaria parasitaemia are presented in Additional file 2. In univariate analysis, HIV-exposed children had a lower odds of parasitaemia compared to HIV-unexposed children (OR = 0.50, p = 0.004). However, after controlling for other variables, HIV exposure status was no longer associated with parasitaemia. In the final multivariate model, enrolment during the high transmission season was associated with a higher odds of parasitaemia (OR = 1.68, p = 0.02), while female gender (OR = 0.66, p = 0.047), older age of mother (OR = 0.81 per five-year increase, p = 0.01), sleeping under a bed net (OR = 0.63, p = 0.03), and having a well-constructed house (OR = 0.25, p = 0.01) were significantly associated with a lower odds of parasitaemia. There was a trend towards lower odds of parasitaemia among children who reported taking TS prophylaxis (OR = 0.20, p = 0.12) but the prevalence of this predictor variable was only 5% and the association did not reach statistical significance. There was no significant association between any of the measures of malnutrition and parasitaemia; stunting (OR = 0.87, p = 0.69), underweight (OR = 1.07, p = 0.86), or wasting (OR = 1.53, p = 0.42).

### Predictor variables associated with malnutrition

The prevalence of stunting, underweight, and wasting were 10%, 7%, and 3%, respectively. Significant associations between predictor variables of interest and measures of malnutrition are presented in Additional file 3. Using multivariate analysis, HIV-exposed children had higher odds of stunting (OR = 2.23, p = 0.005) and wasting (OR = 3.29, p = 0.02) with a trend towards higher odds of being underweight (OR = 1.73, p = 0.09). Children from the poorest households had higher odds of stunting (OR = 2.02, p = 0.01), being underweight (OR = 2.41, p = 0.006), and wasting (OR = 2.66, p = 0.04). None of the other predictor variables of interest were significantly associated with measures of malnutrition.

### Predictor variables associated with moderate-severe anaemia

The prevalence of moderate-severe anaemia (haemoglobin <8 gm/dL) was 11.9%. Significant associations between predictor variables of interest and moderate-severe anaemia are presented in Additional file 4. In univariate analysis, HIV-exposed children had lower odds of anaemia compared to HIV-unexposed children (OR = 0.55, p = 0.004). However, after controlling for other variables, HIV exposure status was no longer associated with anaemia. In the final multivariate model, older infant age (OR = 2.05 per one-month increase, p = 0.004) and parasitaemia (OR = 5.74, p < 0.001) were associated with increased odds of anaemia, with a trend towards higher odds for children who were wasted (OR = 2.85, p = 0.09). Female gender, being from the wealthiest households, and having a well-constructed house were associated with a lower odds of anaemia, however, none of these associations reached statistical significance. There were no significant associations between stunting (OR = 1.38, p = 0.40) or being underweight (OR = 1.84, p = 0.44) and anaemia.

## Discussion

Malaria parasitaemia, measures of malnutrition and moderate-severe anaemia were significant co-morbidities in this study population of HIV-exposed and unexposed Ugandan infants living in a rural area. Interestingly, HIV-exposed infants had lower odds of malaria parasitaemia compared to HIV-unexposed infants. This may be due to the higher use of malaria preventative measures, such as bed nets and TS prophylaxis, in the HIV-exposed group. HIV-exposed children also had a lower prevalence of moderate-severe anaemia. Similarly, this could be explained by the lower prevalence of parasitaemia in the HIV-exposed group, as parasitaemia was strongly associated with moderate-severe anaemia. In contrast, HIV-exposed children had a higher prevalence of measures of malnutrition after controlling for other factors measured in this study. Lower household wealth was also independently associated with a higher prevalence of measures of malnutrition. Also of interest was the finding that having a well-constructed house was independently associated with a significantly lower prevalence of malaria parasitaemia and a trend towards a lower risk of anaemia. These findings provide some evidence of the complex interplay between these important co-morbidities and the factors associated with their prevalence in a novel population of HIV-exposed and unexposed Ugandan infants.

The prevalence of malaria parasitaemia in this study population was 20%, which is considerably lower than the 38% prevalence reported in children <5 years of age from a malaria indicator survey conducted in the same region in 2009 [12]. This difference is probably a reflection of the lower risk of malaria and asymptomatic parasitaemia seen in young infants living in highly malaria-endemic areas due to the placental transfer of antibodies [13]. Indeed, even with the narrow age range of four to six months, older age was significantly associated with a higher prevalence of parasitaemia. Other well-described factors associated with parasitaemia in this study included testing following the rainy season and the protective effect of sleeping under a bed net and the use of TS prophylaxis. Factors associated with parasitaemia in this study that are more difficult to explain include the lower risk seen in females and infants born to older mothers. One of the more interesting findings in this study was the association between living in a well-constructed house and having a lower risk of parasitaemia. Previous studies from Africa have reported that better constructed houses are associated with a lower risk of malaria [14,15]. In addition, a study conducted in western Kenya found that modifying housing structure significantly restricted mosquito entry and thus human exposure to malaria vectors [16]. Other studies have found associations between higher socio-economic status and a lower risk of malaria [15,17] but this association was not observed in this study. Findings have been varied in previous studies evaluating the associations between malnutrition and malaria. Some studies suggest a protective effect against malaria for wasted children [18] and children with stunting [19]. Other studies have found either no association between malnutrition and malaria [20-22] or even an increased risk of malaria among stunted [5,23] and underweight children [4]. In this population of very young HIV negative infants, no association between measures of malnutrition and malaria parasitaemia was observed.

The prevalence of indicators of malnutrition in this population, 10% stunting, 7% underweight and 3% wasted, was somewhat lower than the rates reported for the same area in previous years [6] (17%, 8% and 11%, respectively) and could be explained by the high rates of reported breastfeeding in the population included in this study. Within this novel study population, however, HIV-exposed infants born to HIV-infected mothers had significantly higher risk of malnutrition while controlling for other measured factors. It follows that maternal factors such as breastfeeding and maternal nutrition could be responsible for this observed difference. Poor nutritional status of HIV-infected mothers was suggested in a concurrent clinical trial being conducted at the same location in Uganda, where they were observed to have significantly lower than recommended gestational weight gain [10]. In addition, several other studies conducted in Africa had similar findings of increased prevalence of malnutrition among HIV-exposed but uninfected infants [24-27]. Not surprisingly, children from the poorest households were also at increased risk of malnutrition.

The prevalence of moderate-severe anaemia in this population of young infants was 12%, nearly identical to those rates reported for the region in a malaria indicator survey conducted in 2009 [12]. Many factors were independently associated with an increased risk of anaemia, yet only increasing age of the infant and parasitaemia retained significance in the final statistical model. Increased risk of anaemia with increasing age of the infant correlates to what is seen at the population level in Uganda [12] and could potentially reflect declining nutritional status or the previously discussed increasing risk of malaria parasitaemia in the older infants of this population. What is interesting is that this trend was significant even within the narrow age range (four to six months) of this study population. In addition, there was an observed trend towards decreased risk of anaemia among females, infants in the wealthiest households and those living in a well-constructed house. Wealth and house construction in conjunction with the highly significant increase in odds of anaemia seen with parasitaemia suggest that malaria prevention could have a significant impact on decreasing rates of anaemia in this population. And in fact, previous studies have shown anaemia to be more prevalent among parasitaemic children [28,29] as well as describing the link between wealth and malaria and anaemia [17,30].

The primary limitation of this study was the cross-sectional study design. Associations presented could have been confounded by unmeasured factors and therefore causal inferences cannot be drawn. In addition, the temporal relationship between exposure variables and outcomes of interest cannot be observed. Finally, because this study enrolled participants using convenience sampling and was done in a single geographically defined area, care should be taken in generalizing the results to the other populations.

## Conclusion

Compared to HIV-unexposed infants, HIV-exposed infants had a higher use of malaria preventative measures and lower odds of malaria parasitaemia. Having a better constructed house was also protective against malaria parasitaemia. HIV-exposure was the primary risk factor for measures of malnutrition and the primary risk factor for anaemia was malaria parasitaemia. The findings of this study suggest the need to better target interventions proven to reduce the risk of malaria parasitaemia, such as ITNs and chemoprophylaxis, in addition to the need for exploring new interventions such as improving house construction. With respect to malnutrition, the findings of this study suggest that interventions designed to improve nutritional status should target HIV-exposed children. Finally, data from this study suggest that interventions designed to improve haemoglobin concentrations among young children living in an area of high malaria transmission intensity should focus on reducing the risk of malaria parasitaemia.

## Competing interests

The authors declare that they have no competing interests.

## Authors’ contributions

BO participated in the coordination of the study, data analysis and manuscript preparation. JK participated in coordination of the study, data collection and manuscript preparation. VB, FM and SK participated in the coordination of the study and data collection. MRK participated in the study design and manuscript preparation. GD conceived of the study, participated in its design, coordination, data analysis and manuscript preparation. All authors read and approved the final manuscript.

## Supplementary Material

Additional file 1 Comparison of characteristics between HIV exposed and unexposed infants. Click here for file

Additional file 2 Associations between variables of interest and malarial parasitaemia. Click here for file

Additional file 3 Associations between variables of interest and measures of malnutrition. Click here for file

Additional file 4 Associations between variables of interest and moderate-severe anaemia. Click here for file
