# Supplementary material for: Factors associated with malaria parasitaemia, malnutrition, and anaemia among HIV-exposed and unexposed Ugandan infants: a cross-sectional survey
Source: Malar J. 2012 Dec 27;11:432. doi: 10.1186/1475-2875-11-432 (PMC3544600; doi:10.1186/1475-2875-11-432)
Supplement: Additional file 1 — Comparison of characteristics between HIV exposed and unexposed infants. [file 1475-2875-11-432-S1.docx]

**Table 1. Comparison of characteristics between HIV exposed and unexposed infants**

| **Category** | **Characteristic** | **HIV exposure status** | | **p-value** |
| --- | --- | --- | --- | --- |
|  |  | **Exposed (n=200)** | **Unexposed (n=400)** |  |
| Demographics | Infant’s age in months (SD) | 4.8 (0.7) | 5.4 (0.5) | <0.001 |
|  | Infant female gender, n (%) | 101 (50.5%) | 193 (48.3%) | 0.60 |
|  | Mother’s age in years (SD) | 30.7 (5.7) | 25.5 (6.3) | <0.001 |
|  | Mother the primary care giver, n (%) | 199 (99.5%) | 400 (100%) | 0.16 |
|  | Father deceased, n (%) | 16 (8.0%) | 4 (1.0%) | <0.001 |
| Malaria prevention practices among infants | Reported sleeping under any bednet last night, n (%) | 119 (59.5%) | 170 (42.5%) | <0.001 |
|  | Reported sleeping under an ITN last night, n (%) | 92 (46.0%) | 114 (28.5%) | <0.001 |
|  | Reported currently taking TS prophylaxis, n (%) | 30 (15.0%) | 0 | <0.001 |
| Malaria prevention practices among mothers | Reported sleeping under any bed net last night, n (%) | 120 (60.0%) | 170 (42.5%) | <0.001 |
|  | Reported sleeping under an ITN last night, n (%) | 92 (46.0%) | 114 (28.5%) | <0.001 |
|  | Reported sleeping under a bed net during last pregnancy, n (%) | 144 (72.0%) | 223 (55.8%) | <0.001 |
|  | Reported sleeping under an ITN during last pregnancy, n (%) | 99 (49.5%) | 140 (35.0%) | 0.001 |
|  | Reported taking TS prophylaxis during last pregnancy, n (%) | 181 (90.5%) | 0 | <0.001 |
|  | Reported taking SP prophylaxis during last pregnancy, n (%) | 110 (55.0%) | 325 (81.3%) | <0.001 |
|  | Reported currently taking TS prophylaxis, n (%) | 188 (94%) | 0 | <0.001 |
| Current breastfeeding practice | Not breastfeeding, n (%)  Partial breastfeeding, n (%)  Predominant breastfeeding, n (%)  Exclusive breastfeeding, n (%) | 0  98 (49.0%)  6 (3.0%)  96 (48.0%) | 1 (0.3%)  315 (78.8%)  36 (9.0%)  48 (12.0%) | <0.001 |
| Household wealth index | Lowest tertile  Middle tertile  Highest tertile | 66 (33.0%)  62 (31.0%)  72 (36.0%) | 131 (32.8%)  143 (35.8%)  126 (31.5%) | 0.43 |
| House structure | Well-constructed house* | 40 (20.0%) | 29 (7.3%) | <0.001 |

***** Iron sheets on roof, burnt brick or cement walls, and cement floor

ITN = Insecticide-treated net; TS = trimethoprim-sulphamethoxazole; SP = sulphadoxine-pyrimethamine
