# Supplementary material for: Factors associated with malaria parasitaemia, malnutrition, and anaemia among HIV-exposed and unexposed Ugandan infants: a cross-sectional survey
Source: Malar J. 2012 Dec 27;11:432. doi: 10.1186/1475-2875-11-432 (PMC3544600; doi:10.1186/1475-2875-11-432)
Supplement: Additional file 2 — Associations between variables of interest and malarial parasitaemia. [file 1475-2875-11-432-S2.docx]

**Table 2. Associations between variables of interest and malarial parasitaemia**

|  | **Prevalence of parasitaemia** | |  | |  | |
| --- | --- | --- | --- | --- | --- | --- |
| **Variables of interest** | **Variable present** | **Variable not present** | **Univariate analysis** | | **Multivariate analysis** | |
|  |  |  | **OR (95% CI)** | **p-value** | **OR (95% CI)** | **p-value** |
| HIV exposed | 27/200 (13.5%) | 95/400 (23.8%) | 0.50 (0.31-0.80) | 0.004 | Not included in final model | |
| Enrolled Dec 2010 – Mar 2011 | 59/230 (25.7%) | 63/370 (17.0%) | 1.68 (1.13-2.51) | 0.01 | 1.68 (1.11-2.56) | 0.02 |
| Infant’s age (per one month increase) | N/A | | 1.32 (0.96-1.82) | 0.09 | Not included in final model | |
| Infant female gender | 50/294 (17.0%) | 72/306 (23.5%) | 0.67 (0.45-1.00) | 0.05 | 0.66 (0.43-0.99) | 0.047 |
| Mother’s age (per five year increase) | N/A | | 0.74 (0.63-0.88) | <0.001 | 0.81 (0.69-0.96) | 0.01 |
| Infant reported sleeping under a bednet last night | 45/289 (15.6%) | 77/311 (24.8%) | 0.56 (0.37-0.84) | 0.006 | 0.63 (0.41-0.97) | 0.03 |
| Infant reported currently taking TS prophylaxis | 1/30 (3.3%) | 121/570 (21.2%) | 0.13 (0.02-0.95) | 0.04 | 0.20 (0.03-1.51) | 0.12 |
| Well-constructed house | 4/69 (5.8%) | 118/531 (22.2%) | 0.22 (0.08-0.60) | 0.003 | 0.25 (0.09-0.72) | 0.01 |

TS = trimethoprim-sulphamethoxazole
