# Supplementary material for: Factors associated with malaria parasitaemia, malnutrition, and anaemia among HIV-exposed and unexposed Ugandan infants: a cross-sectional survey
Source: Malar J. 2012 Dec 27;11:432. doi: 10.1186/1475-2875-11-432 (PMC3544600; doi:10.1186/1475-2875-11-432)
Supplement: Additional file 3 — Associations between variables of interest and measures of malnutrition. [file 1475-2875-11-432-S3.docx]

**Table 3. Associations between variables of interest and measures of malnutrition**

| **Variables of interest** | **Prevalence of stunting** | | **Univariate analysis** | | **Multivariate analysis** | |
| --- | --- | --- | --- | --- | --- | --- |
|  | **Variable present** | **Variable not present** | **OR (95% CI)** | **p-value** | **OR (95% CI)** | **p-value** |
| HIV-exposed | 29/200 (14.5%) | 31/400 (7.8%) | 2.02 (1.18-3.46) | 0.01 | 2.23 (1.28-3.87) | 0.005 |
| Infant reported sleeping under a bed net last night | 22/289 (7.6%) | 38/311 (12.2%) | 0.59 (0.34-1.03) | 0.06 | 0.59 (0.33-1.06) | 0.08 |
| Lowest tertile for household wealth index | 30/197 (15.2%) | 30/403 (7.4%) | 2.23 (1.30-3.82) | 0.003 | 2.02 (1.16-3.52) | 0.01 |
| **Variables of interest** | **Prevalence of underweight** | | **Univariate analysis** | | **Multivariate analysis** | |
|  | **Variable present** | **Variable not present** | **OR (95% CI)** | **p-value** | **OR (95% CI)** | **p-value** |
| HIV-exposed | 19/200 (9.5%) | 23/400 (5.8%) | 1.72 (0.91-3.24) | 0.09 | 1.73 (0.91-3.27) | 0.09 |
| Lowest tertile for household wealth index | 22/197 (11.2%) | 20/403 (5.0%) | 2.41 (1.28-4.53) | 0.006 | 2.41 (1.28-4.55) | 0.006 |
| **Variables of interest** | **Prevalence of wasting** | | **Univariate analysis** | | **Multivariate analysis** | |
|  | **Variable present** | **Variable not present** | **OR (95% CI)** | **p-value** | **OR (95% CI)** | **p-value** |
| HIV-exposed | 11/200 (5.5%) | 7/400 (1.8%) | 3.27 (1.25-8.56) | 0.02 | 3.29 (1.25-8.66) | 0.02 |
| Lowest tertile for household wealth index | 10/197 (5.1%) | 8/403 (2.0%) | 2.64 (1.03-6.80) | 0.04 | 2.66 (1.03-6.90) | 0.04 |
