# Supplementary material for: Factors associated with malaria parasitaemia, malnutrition, and anaemia among HIV-exposed and unexposed Ugandan infants: a cross-sectional survey
Source: Malar J. 2012 Dec 27;11:432. doi: 10.1186/1475-2875-11-432 (PMC3544600; doi:10.1186/1475-2875-11-432)
Supplement: Additional file 4 — Associations between variables of interest and moderate-severe anaemia. [file 1475-2875-11-432-S4.docx]

**Table 4. Associations between variables of interest and moderate-severe anaemia**

| **Variables of interest** | **Prevalence of anaemia*** | | **Univariate analysis** | | **Multivariate analysis** | |
| --- | --- | --- | --- | --- | --- | --- |
|  | **Variable present** | **Variable not present** | **OR (95% CI)** | **p-value** | **OR (95% CI)** | **p-value** |
| HIV exposed | 16/199 (8.0%) | 55/399 (13.8%) | 0.55 (0.30-0.98) | 0.04 | Not included in final model | |
| Enrolled Dec 2010 – Mar 2011 | 34/229 (14.9%) | 37/369 (10.0%) | 1.56 (0.95-2.57) | 0.08 | Not included in final model | |
| Infant’s age (per one month increase) | N/A | | 2.11 (1.35-3.31) | 0.001 | 2.05 (1.27-3.32) | 0.003 |
| Infant female gender | 26/294 (8.8%) | 45/304 (14.8%) | 0.56 (0.33-0.93) | 0.03 | 0.61 (0.35-1.06) | 0.08 |
| Mother’s age (per five year increase) | N/A | | 0.83 (0.69-1.02) | 0.08 | Not included in final model | |
| Infant reported sleeping under an ITN last night | 18/206 (8.7%) | 53/392 (13.5%) | 0.61 (0.35-1.08) | 0.09 | Not included in final model | |
| Exclusive breastfeeding | 11/144 (7.6%) | 60/454 (13.2%) | 0.54 (0.28-1.06) | 0.08 | Not included in final model | |
| Highest tertile for household wealth index | 16/197 (8.1%) | 55/401 (13.7%) | 0.56 (0.31-1.00) | 0.05 | 0.59 (0.32-1.11) | 0.10 |
| Well-constructed house | 1/69 (1.5%) | 70/529 (13.2%) | 0.10 (0.01-0.70) | 0.02 | 0.18 (0.02-1.33) | 0.09 |
| Parasitaemia | 39/122 (32.0%) | 32/476 (6.7%) | 6.52 (3.86-11.0) | <0.001 | 5.74 (3.34-9.87) | <0.001 |
| Wasted | 5/18 (27.8%) | 66/580 (11.4%) | 3.00 (1.03-8.67) | 0.04 | 2.85 (0.85-9.54) | 0.09 |

* Anaemia defined as hemoglobin < 8 gm/dL (results missing for two study participants)

ITN = Insecticide-treated net
